# Industrial IoT Monitoring: Technologies and Architecture Proposal

**DOI:** 10.3390/s18103568

**Published:** 2018-10-21

**Authors:** Duarte Raposo, André Rodrigues, Soraya Sinche, Jorge Sá Silva, Fernando Boavida

**Affiliations:** 1 Centre of Informatics and Systems of the University of Coimbra, Coimbra 3030-290, Portugal; arod@dei.uc.pt (A.R.); smaita@dei.uc.pt (S.S.); sasilva@dei.uc.pt (J.S.S.); boavida@dei.uc.pt (F.B.); 2 Polytechnic Institute of Coimbra, ISCAC, Coimbra 3040-316, Portugal; 3 DETRI, Escuela Politécnica Nacional, Quito 170517, Ecuador

**Keywords:** monitoring, industrial IoT, industrial WSN, WirelessHART, ISA100.11a, WIA-PA, ZigBee, Industry 4.0

## Abstract

Dependability and standardization are essential to the adoption of Wireless Sensor Networks (WSN) in industrial applications. Standards such as ZigBee, WirelessHART, ISA100.11a and WIA-PA are, nowadays, at the basis of the main process-automation technologies. However, despite the success of these standards, management of WSNs is still an open topic, which clearly is an obstacle to dependability. Existing diagnostic tools are mostly application- or problem-specific, and do not support standard-based multi-network monitoring. This paper proposes a WSN monitoring architecture for process-automation technologies that addresses the mentioned limitations. Specifically, the architecture has low impact on sensor node resources, uses network metrics already available in industrial standards, and takes advantage of widely used management standards to share the monitoring information. The proposed architecture was validated through prototyping, and the obtained performance results are presented and discussed in the final part of the paper. In addition to proposing a monitoring architecture, the paper provides an in-depth insight into metrics, techniques, management protocols, and standards applicable to industrial WSNs.

## 1. Introduction

The Internet of Things (IoT) currently makes it possible to have a world sensed by and connected to all kinds of devices. Wireless Sensor Network (WSN) technology is the key for connecting physical and virtual environments. This technology is growing up so rapidly that in 2011 Cisco-IBSG estimated that globally there would be 50 billion interconnected “things” by 2020 [1]. The IoT paradigm leads to an extremely large number of new opportunities and technical challenges in several fields in general, and in the industrial field in particular. In industry, wired technologies continue to be prevalent [2,3]. Digital technologies like ModBus, ProfiBus, CanBus, HART [4], and even analogue technologies like 4–20 mA [5], are used to monitor and control most processes. Despite the high reliability of such technologies, proven over many years, wired technologies are expensive, difficult to install, time consuming, and unable to cope with the requirements of Cyber Physical Systems (CPS) and Industry 4.0. In Industry 4.0, CPSs will confer micro intelligence (namely processing and networking capabilities) to industrial objects, reducing even further today’s already short production cycles [6]. Additionally, in the near future, new markets will drive CPSs to increase their level of adaptability, directly connecting customers to manufacturing facilities, using Cyber Physical Manufacturing Systems (CPMS) [7,8]. Thus, WSNs or, more specifically, Industrial Wireless Sensor Networks (IWSN), are fundamental for meeting the requirements of Industry 4.0.

IWSN characteristics like low operating costs, self-organization, self-configuration, flexibility, rapid-deployment, and easy upgrading, make them ideal to industrial scenarios. However, despite all of these favourable characteristics, the adoption of WSNs in industry requires standards, dependability, ease of use, network security, extended battery life, low cost, and IP connectivity [9]. In recent years, considerable effort was made in order to design technologies that meet these requirements, and to standardize IWSN technology. Standards like IEEE 802.15.4 [10] and IEEE 802.15.1 [11] are the technology foundation of many industrial applications for process and factory automation. IEEE 802.15.4 is the base technology for standards such as ZigBeePRO [12], WirelessHART [13], ISA100.11a [14], and WIA-PA [15]. These are widely used in process automation applications in the areas of chemical manufacturing, pulp and paper, oil and gas, and glass and mineral [16]. On the other hand, IEEE 802.15.1 is the base technology for standards such as WISA [17] and WSAN-FA [9], widely used in factory automation applications in the areas of assembly process for automotive, consumer products and electronics [18].

Nevertheless, although standards compliance is necessary, it is not enough to guarantee ISWN reliability per se. Sensor node components, either at hardware or firmware levels, and the network itself, can be at the root of a variety of faults [19]. Sensor nodes are inherently resource-constrained devices in terms of energy, processing power and memory capacity. In this respect, the Internet Engineering Task Force (IETF) recently defined three classes of devices [20]: Class 0 for devices with less than 10 KB of RAM and 100 KB of flash memory; Class 1 for devices with around 10 KB of RAM and 100 KB of flash; and Class 2 for devices that have more resources but are still quite constrained when compared to high-end devices. In addition to the device constraints mentioned, sensor, network, and application heterogeneity lead to extremely complex IWSN solutions and, consequently, to fault-proneness. These characteristics impose adequate, carefully-designed strategies in the development of sensor nodes firmware, hardware architectures, and operating systems (OSs) kernel (e.g., choosing between exokernel, microkernel, monolithic approach or hybrid approach) [21]. As a way of example, OEM manufacturers can build their products based on a single chip (comprising wireless communications and processing capabilities) [22], or separate microcontroller and radio (connected by SPI or UART) [23]. Furthermore, applications may be developed on “bare metal” (which makes them very hardware-specific), or using one of the available OSs (e.g., Contiki [24], RIOT [25], FreeRTOS [26]). Another important component is the network. For instance, despite the inclusion of security mechanisms in all of the referred standards, there are known attacks on WirelessHART, ZigBeePRO, ISA100.11a, and WIA-PA [27,28,29,30]. Additionally, some of these technologies, namely WirelessHART, ZigBeePRO, and WIA-PA, are not immune to interference from equipment complying with other standards, such as IEEE 802.11, when operating in the ISM 2.4 Ghz frequency band. Such problems may lead to early sensor node energy depletion, and subsequent replacement, increasing the costs of network operation. Because of this, post-deployment tools are needed in order to adequately monitor IWSNs, thus contributing to the global system reliability.

In the last decade, a wide range of WSN post-deployment tools [31] were developed. Some of them can improve the reliability of WSN by detecting network, firmware, and/or hardware problems. These tools help developers in both deployment and post-deployment environments by making several firmware- and hardware-related metrics accessible, and by detecting problems, using, for instance, sniffers or sink nodes. However, despite the effort to build such tools, most of them were designed for specific applications, require specific or dedicated hardware, consume non-negligible amounts of energy, do not implement security mechanisms, are complex to configure and/or to use, and do not allow the centralized management of multiple industrial standards like ZigBeePRO, WirelessHART, ISA100.11a, and WIA-PA.

In this context, the contributions of this paper are the following: (i) a survey of ISWN standards (for process-automation) and their features per OSI layer; (ii) an overview of the reports and metrics made available by each of the IWSN standards; (iii) a survey of the main techniques for WSN monitoring at network, firmware, and hardware levels; (iv) a proposal for an industrial IoT monitoring architecture that builds on IEEE 802.15.4-based industrial standards (WirelessHART, ISA100.11a, ZigBeePRO and WIA-PA) to provide management functionality; and (v) a proof-of-concept implementation of the proposed architecture. It should be noted that the proposed architecture does not increase the cost of node manufacturing, has negligible impact on the main sensor node application, hardware components, and network bandwidth and, last but not least, does not significantly increase energy consumption.

The remainder of this paper is organized as follows. Section 2 details the main industrial IoT technologies, and provides a revision of applicable management protocols. In Section 3, representative diagnostic tools for collecting hardware, firmware and network metrics are described. Section 4 presents the proposed architecture in detail and, at the same time, explains how technologies, approaches, and protocols can be used to implement it. Section 5 presents a proof-of-concept implementation in a testbed using the WirelessHART standard, with the aim of assessing the architecture’s impact in terms of energy consumption, application latency, RAM and flash utilization, and network traffic. Section 6 compares the proposed architecture with related work in this domain. Lastly, Section 7 presents the conclusions and guidelines for future work.

## 2. Technologies for Industrial IoT

Reliable, controlled operation, and use of standardized technologies are key to the adoption of WSNs in industrial applications [32]. In best-effort type networks, all devices in the network obtain an unspecified data rate and delivery time, depending on the traffic load [33]. As a result, data is delivered without any quality of service guarantees. However, in general, industrial process automation applications have stringent latency requirements. For instance, monitoring applications should guarantee an average latency of 100 ms; control applications should guarantee 10 ms to 100 ms latency; and, lastly, safety applications should guarantee a maximum latency of 10 ms [14]. To meet these requirements, IWSNs may have to pre-allocate network bandwidth and physical resources, thus avoiding statistical effects that lead to insufficient bandwidth, uncontrolled jitter, and congestion losses. With these constraints in mind, this section surveys the main IWSN technologies and standards (namely, IEEE 802.15.4, ZigBeePRO WirelessHART, ISA100.11a and WIA-PA), highlighting their main features, monitoring functionality, and applicable management protocols.

### 2.1. IWSN Standards

This subsection analyses the characteristic features of each of the main IWSN standards, namely ZigBeePRO, WirelessHART, ISA100.11a, and WIA-PA. The analysis is done on a per-OSI-layer basis, starting with the physical layer and working up to the application layer. Table 1, below, presents a summary of the referred features, and may be used as guidance for the reader.

IEEE 802.15.4 [10] is a standard for low-rate wireless personal area networks (LR-WPANs) that specifies the physical (PHY) and medium access control (MAC) layers. These layers’ design was optimized for very low-power consumption, high reliability (using mesh networks), low-data rates, and low-cost. ZigBee, WirelessHART, ISA100.11a, and WIA-PA have all adopted IEEE 802.15.4 at the PHY layer. However, because WirelessHART, ISA100.11a, and WIA-PA target worldwide adoption, they’ve chosen to use the 2.4 GHz frequency band only, as opposed to ZigBee, which can use the 868 Mhz and 915 Mhz bands as well [18].

At the MAC layer, there are significant differences in the adoption of the IEEE 802.15.4 by each of the four standards. At this layer, networks can work in beacon or non-beacon modes of operation. When using the beacon mode, sensor nodes receive a specific message that is used to synchronize the network devices and, at the same time, to identify the network, and to describe the structure of the frame. Beacon networks are capable of detecting other beacon networks, and, for this reason, beacon networks can coexist in the same geographical area. Additionally, IEEE 802.15.4 uses a superframe structure in order to manage nodes channel access. The superframe is formed by three different parts: Contention Access Period (CAP), Contention Free Period (CFP), and inactive period. This superframe structure is used in ZigBee and WIA-PA because these two standards operate in beacon mode. On the other hand, WirelessHART and ISA100.11a do not operate in beacon-mode, as their developers considered that this mode is not good enough for industrial applications (Note: WIA-PA also shares the same view; however, its authors opted for maintaining full compatibility with IEEE 802.15.4-based networks, and implement additional features at the Data Link (DL) layer). As a result, WirelessHART and ISA100.11a implemented their own superframes [34]. WirelessHART’s superframe is composed of 10 ms timeslots, while ISA100.11a can operate in any of three superframe modes (short, long, and hybrid). Finally, ZigBee can use a slotted or unslotted CSMA/CA mechanism for managing the access to the wireless medium, while the remaining standards (WirelessHART, ISA100.11a and WIA- PA) use TDMA and CSMA, providing a high and medium level of latency determinism, respectively.

Neither WirelessHART or ISA100.11a implement the full IEEE 802.15.4 MAC layer, as they consider that the MAC layer of IEEE 802.15.4 is not capable of delivering the deterministic latency needed by industrial applications. As such, these standards extend and complement medium access mechanisms with functionality at the data link (DL) layer [9], namely, Time Synchronized Channel Hopping (TSCH), which then evolved to the new IEEE 802.15.4.e standard. The TSCH mechanism offers two significant improvements: the possibility to have deterministic latency (communication resources are pre-allocated); and a mechanism of channel hopping that minimizes interference with nearby devices that operate in the same frequency, such as IEEE 802.11 devices. On the other hand, the WIA-PA follows the IEEE 802.15.4 standard at the DL layer, including functionalities like time synchronization and frequency hopping techniques. Lastly, ZigBee does not implement any mechanism at this layer. It is also worthwhile mentioning that, in addition to extending/complementing MAC layer functionality, the DL layer is also used by ISA100.11a for implementing some network-related functions, specifically in what concerns routing. In fact, this standard implements two types of routing: one at the DL layer, which handles all the IEEE 802.15.4 traffic, and another one at the Network (NWK) layer, responsible for handling the IPv6 backbone traffic, as we will see below.

At the NWK layer, the choice of supported functionality and routing protocols is often influenced by the network architectures [18]. For instance, ZigBee offers the possibility of having star, tree, and mesh topologies, and defines several field devices: coordinator, routers, and end-devices. Tree routing and Z-AODV protocols are used when the network operates in tree or mesh topology, respectively. Despite the fact that mesh networks can be considered more reliable, in ZigBee, the utilization of this topology is not suitable for industrial applications, due to the overhead and non-deterministic latency of on-demand protocols like Z-AODV [9]. In the case of WirelessHART, the basic network devices are: field devices, gateway, access points, and network and security manager. Typically, WirelessHART gateways support the role of security and network manager, and access point [35].

WirelessHART networks may operate in star or mesh topologies. However, the mesh topology is the most used one, due to its flexibility, inherent fault-tolerance, and ease of deployment and configuration. With this topology, routing can be done by using graph routing or source routing. When graph routing is used, the network manager needs to compute all graphs in the network and share them with sensor nodes. All communications using graph routing always consider two different paths to ensure reliability. In contrast, when source routing is used, network packets are forward between intermediate devices without the need for prior route information (the path of the packet is specified in the packet itself) [3]. Differently from the other standards, ISA100.11a specifies two types of networks: IEEE 802.15.4 (i.e., WSN) network, and backbone network. In the WSN network, ISA100.11a defines three device types: routing devices, field devices, and handheld devices. The routing mechanisms available in this network are the same as the ones in WirelessHART. Additionally, in the infrastructure (i.e., backbone) side, ISA100.11a uses 6LowPAN, thus allowing for direct communication between external IP devices and ISA100.11a devices. Lastly, WIA-PA supports a hierarchical topology that uses star and mesh, or star-only topology. In the case of the mesh topology, the network operates using routers and gateways, while in the star topology the network is composed of routers and field/handheld devices. At this level, field devices are cluster members that acquire sensor information and send it to the cluster heads (routers). Then, the cluster-heads form the mesh network. Each routing device in the network shares its neighbour information with the network manager, and then the network manager computes and shares the static routes. For each pair of devices that want to communicate, at least two routing paths are assigned.

The transport (TP) layer is responsible for providing host-to-host communication services between applications. As can be seen in Table 1, only WirelessHART and ISA100.11a implement data transport functions at this layer, supporting different service level agreements. Optionally, ISA100.11a offers end-to-end security at this layer. In contrast to ISA100.11a and WirelessHART, WIA-PA provides different service level agreements at the application sub-layer (APS), and not at the transport layer. The service-level agreements available in each standard are presented in Section 4 and Section 4.2. Last but not least, ZigBee does not support any transport layer functionality nor does it support traffic differentiation at the APS sub-layer. Moreover, in ZigBee, fragmentation, reassembly, and device discovery are implemented at the APS sub-layer.

The application (APP) layer is the layer at which the connection between legacy systems and IEEE 802.15.4-based systems takes place [9,18]. At this layer, there are two important options that can be identified. WirelessHART uses a command-oriented approach; alternatively, ISA100.11a, ZigBee, and WIA-PA use a more flexible object-oriented approach. Object-oriented approaches are more flexible than command-oriented approaches because they allow for protocol translation by mapping attributes from one protocol to the other. As for native applications, ZigBee supports the ZigBee profiles; WirelessHART supports the HART protocol; WIA-PA supports native protocols like Profibus, FF, and HART; last but not least, ISA100.11a supports the ISA100.11 application protocol.

### 2.2. IWSN Reports

IWSN standard technologies make device and network state information available to a variety of entities, e.g., network neighbours or a central management device. In general, this information is shared between nodes to compute routes, allocate bandwidth, calculate link costs between network devices, or generate alarms when critical events occur (e.g., link failure, route failure, low battery level, etc.). This subsection presents the network and data link layer reports available in each of the standards being considered in this paper [12,13,14,15], and describes the context in which the reports are used. Table 2 presents a summary of the referred reports, and may be used as guidance for the discussion.

The ZigBee standard comprises three report types that are used for sharing network- and node-related information: (1) link status report; (2) network status report, and (3) network report. Link status reports share sensor node’s neighbours incoming and outgoing link costs. The report is broadcasted by ZigBee coordinators and routers in one-hop fashion. This report is useful during network discovery, to find neighbour devices, and in the operation phase for updating the devices’ neighbour table. Network status reports are sent by devices in order to report errors and other events that arise at the network layer. The report can be sent in unicast or broadcast modes over the network, and can only pertain to an event at a time. The list of the possible reported events is presented in the Table 2. Last but not least, network reports allow a device to report network events, like PAN conflict, or the radio channel condition to the coordinator. As limitation, in the tree topology, some of these packets nay not be received by the ZigBee coordinator, due to the presence of routing devices, that make their own routing decisions. On the other hand, when using a star topology, all the packets will be received at the ZigBee coordinator.

Contrary to what happens in ZigBee, in WirelessHART, the network manager controls all communication in the network, and only authorizes new services if resources are available. Consequently, field devices (here with full network capabilities) must share node state and network based information with the network manager. In WirelessHART, all network reports arrive to the network manager by using the maintenance service. WirelessHART specifies four types of reports: (1) device health; (2) neighbour health list; (3) neighbour signal levels; and (4) alarm report. Device health reports summarize all the communication statistics of a unique field device and are periodically sent to the network manager. The statistics include generated packets by device, terminated packets by device, power status, and others. Neighbour health list reports include statistics about the communication with all neighbours linked to a field device. These reports include the total number of linked neighbours, the mean Received Signal Level (RSL) to the neighbour, and packets and errors statistics. Neighbour signal level reports include statistics of discovered but not linked neighbour devices detected by a field device. When a device wants to connect to the network, it usually sends a join request and a neighbour signal level report. Lastly, WirelessHART defines several alarm types: path down alarm; source route failed alarm; and graph route failed alarm.

Similarly to what happens in WirelessHART, in ISA100.11a networks, the system manager controls all communication in the network. However, in this standard, WSN routing takes place at the DL layer, due to the use of 6LoWPAN at the network layer. Network metrics in ISA100.11a are shared at the MAC layer, instead of being shared at the NWK layer. ISA100.11a defines two groups of network reports: (1) connectivity alert, and (2) neighbour discovery alert. Connectivity alerts comprise two types of reports: per-neighbour report and per-channel report. Per-neighbour reports contain neighbours’ connection statistics. On the other hand, per-channel reports contain per-channel all-neighbours statistics, and convey them to the system manager. Finally, neighbour discovery alerts are sent periodically to the system manager with a list of overheard neighbours. The system manager makes new routing decisions based on these reports. Per-neighbour reports, per-channel reports, and neighbour discovery alert, are similar to WirelessHART’s neighbour health list, device health, and neighbour signal level, respectively.

Last but not least, in WIA-PA, route computation is also performed by the network gateway, similarly to WirelessHART and ISA100.11a. WIA-PA defines four types of reports: (1) device status report, (2) channel condition report, (3) neighbour report, and (4) path failure report. Device status reports include statistics about the condition of field devices and routers, such as the number of packets exchanged with neighbours, number of restarts, and uptime. Channel condition reports send statistics grouped by channel and neighbour to the network gateway. These statistics include link quality, packet loss rate, and number of retries. Neighbour reports, also received by the network gateway, group neighbour statistics and neighbour scheduling details, such as backoff counter and exponent, transmitted and received packets, and number of acknowledgments. Lastly, path failure reports are generated when a route path failure occurs. These reports are sent by a routing device to the network gateway, whenever the retransmission counter of a specific path exceeds a given threshold.

### 2.3. Management Protocols

Network management emerged in traditional networks with the need to control and monitor networks as they grew up in size and complexity. ISO/IEC 7498-4 [36] was one of the first initiatives to establish a management framework, by defining a set of functional areas, namely: fault, configuration, accounting, performance, and security management. Furthermore, ISO/IEC 7498-4 proposed the use of managed objects as abstractions for network resources, which, in turn, contain several attributes. In current protocols, managed objects can be defined using several syntaxes, like SMIv2 [37], YANG [38], and XML [39]. Managed objects data are stored in management databases and accessed by management protocols. In this sub-section, we identify two types of management protocols: (1) protocols designed for traditional networks; and (2) protocols designed for networks of resource-constrained devices, that can also be used in some traditional networks.

As one of most used protocols for the management of IP devices, SNMP [37] provides most of the basic functionality defined in ISO/IEC 7498-4. The standard specifies an architecture based on agents and managers. Devices being monitored, run SNMP agents that share the device state information with an SNMP manager, by using query/response interactions and trap notifications. Each SNMP agent has a collection of managed objects whose data are stored in a Management Information Base (MIB). Each object is identified using a specific object identifier (OID). Despite the success of SNMP for monitoring purposes, the protocol failed to provide an effective and reliable way to configure devices. Thus, IETF started a working group that wrote an informational RFC [40] with several requirements that coming network management standards should implemented. This work was the basis for the NETCONF [41] protocol. Differently from SNMP’s manager-agent architecture, NETCONF uses a client-server architecture. The server runs on a management device (SNMP agent) and shares the monitoring information (also by query/response and notification messages) with the client (SNMP manager). Additionally, when devices need to be configured, the client may send several configuration commands in one or several transactions. The transactions supported in NETCONF give operators the capability of sending out-of-order commands, and of performing rollback and commit operations.

The management protocols mentioned up to now were designed for traditional networks. In [42], the authors implemented traditional network management protocols in networks of constrained devices, and concluded that SNMP and NETCONF are not suitable for this type of networks. For instance, the use of TLS encryption in NETCONF adds significant overhead in terms of session time (i.e., in the order of seconds). In later years, with the growth of IoT, new solutions that address resource-constrained devices and 6LowPAN networks were proposed. These solutions also take advantage of transport protocols specially designed for resource-constrained devices, like CoAP [43].

One of the first solutions developed with a focus on the management of 6LowPAN networks was the LoWPAN Network Management (LNMP) protocol [44]. The LNMP protocol implements a solution based on SNMP that targets 6LowPAN networks. In this solution, 6LowPAN gateways convert the information from 6LowPAN networks to SNMP MIBs and make it available over IP. On the other hand, other management architectures were evaluated, and new research directions that take advantage of new technologies like HTTP appeared. In [45], the authors evaluated the use of different architectures like SNMP, Resource-Oriented Architecture (ROA), and Service-Oriented Architecture (SOA), and concluded that ROA architectures are more suitable for resource-constrained devices in terms of response time and power consumption, and are less sensitive to changes in timeout. As a result, a RESTful version of NETCONF was created, named RESTCONF [46]. Distinct from NETCONF, which uses SSH and TCP, RESTCONF allows the communication between server and client using HTTP operations. Using HTTP at the application layer enables clients to receive notifications without maintaining a permanent connection with the server, as it is the case of NETCONF (one of its major drawbacks). The syntax used in RESTCONF is YANG, the same syntax used in NETCONF. Also using an ROA-based architecture but with a different application protocol, the CoAP Management Interface [47] (COMI) is an internet draft that intends to provide access to resources specified in YANG or SMIv2, using CoAP. The draft defines, as in the cases of NETCONF and RESTCONF, a separation between operational and configuration data store, the use of DTLS, and a conversion of YANG string identifiers to numeric identifiers that contributes to reducing the payload size.

Last but not least, LwM2M [39] is also a protocol designed for resource-constrained devices. This protocol provides device management and application management, an aspect that differs from the previously mentioned network management protocols. As COMI, LwM2M also supports the use of CoAP at the application layer. Being a REST-based protocol, LwM2M uses GET/PUT/POST operations to perform read/write/execute operations over the managed objects resources. In this protocol, the definition of managed objects is done using XML. An extensive list of managed objects is available in OMA [48]. In addition to these, OMA allows the creation of specific objects by individuals, organizations, and companies.

Summing up, in this sub-section, a set of network management protocols were presented (see Table 3). By analysing the protocols presented here, we identified protocols suitable to managing traditional networks (switches, routers, computers), and protocols designed for networks of resource-constrained devices (e.g., sensor nodes). An important trend is that new protocols like LwM2M use general-purpose languages to describe the managed objects (e.g., XML), instead of YANG and SMIv2. All of these management protocols can be incorporated into the different gateways identified in the industrial standards presented before (i.e., ZigBee coordinator, WirelessHART network manager, ISA100.11a system manager, and WIA-PA network gateway) because these roles are performed by unconstrained devices. Finally, protocols like SNMP, NETCONF, RESTCONF, COMI and LwM2M cannot be directly used in sensor nodes because current standard industrial technologies do not allow running application protocols other than their own. Thus, the management of these kinds of networks can only be done in a standardized way at gateway level. Only new standards like 6tisch [49] support the management of sensor node devices using COMI or LwM2M because 6tisch uses the CoAP protocol at the application level. Nevertheless, due to the fact that no 6tisch-based products are available and, consequently, it is not yet used in industrial settings, 6tisch will not be addressed in this paper.

## 3. WSN Diagnostic Tools

The design of a diagnostic tool applicable to low-end IoT devices, like WSN nodes, is a tough task due to their characteristics and diversity. WSN nodes have limited resources, support a variety of application architectures, and rely on complex network mechanisms. In addition, WSN applications can be developed for a specific operating system, or even almost from scratch. These characteristics make it difficult, or even impossible, to develop a common diagnostic tool for all kinds of scenarios, and, at the same time, compatible with several operating systems. Despite this, in the last decade, several diagnostic tools were proposed in order to provide inside views on WSN networks’ and nodes’ behaviour. In [31], the authors analyse an extensive set of diagnostic tools. In this section, some of the tools described in [31], as well as more recent tools like [50,51,52,53,54], will be presented from a diagnostic target perspective, organizing their presentation into network-based, firmware-based, and hardware-based tools.

### 3.1. Network Tools

The nature of wireless communications makes this technology unreliable and failure-prone. In this context, diagnostic tools that are able to collect network metrics and evaluate the state of the network are essential. Three types of approaches can be used to collect network information: (1) passive, using extra hardware to collect network information without interference; (2) active, using on-node available resources; and (3) hybrid, using a mix of active and passive approaches.

When passive approaches are used, the acquisition of network-related information is made by installing extra sniffer nodes or sniffer capabilities in the existing sink nodes. These diagnostic tools may differ in the type of sniffer nodes, in the storage approach, and in the way the gathered information is transmitted. Specifically, some of the existing solutions use sink sniffer nodes to collect traffic data from the network; others use extra networks of sniffers deployed with the main WSN; others use sniffer nodes that collect data and store it in memory; and, lastly, the most expensive in terms of network installation, send network-related information by wired technologies (e.g., SNIF [55], SNTS [56], PDA [57], LiveNet [58] and L-SNMS [59]).

Differently from passive tools that rely on extra hardware to monitor the network, active approaches use on-node metrics already available in sensors nodes. Active tools are easy to install, do not need extra hardware and, consequently, are less expensive when compared to passive approaches. However, active tools may have impact on node resources, as memory, energy, processing, and network throughput are needed to collect, store, and transport network-related information. Some of the tools gather traffic metrics from the sensor nodes’ operating system, others from the network layer, and others directly from sink nodes, based on the received network traffic. In addition, there is a specific set of tools that use software scripts deployed in sensor nodes to collect statistics about the network traffic seen by the node. Subsequently to data acquisition, the transport of network-related information can be made using the main application channel or a secondary channel. To minimize the impact of data transport, some tools use compression and aggregation techniques so as to reduce the traffic overhead. Examples of this type of tools are Marionete [60], Megs [61], Memento [62], Wringer [63], 6PANview [50], and D2 [53].

Lastly, hybrid approaches use a mix of methods described in the cases of the active and passive approaches. Examples of this type of tools are Sympathy [64] and Dustminer [65].

### 3.2. Firmware Tools

Another source of faults commonly addressed by diagnostic tools is the sensor nodes’ firmware. After firmware development, sensor nodes are deployed in the field and, in some cases, faults may stay in a dormant state until an input activates them. If the error is not properly handled, a firmware failure may occur and sensor node data may not be delivered or may be corrupted. In an extreme although not uncommon situation, a firmware fault may even prevent a sensor node from entering sleep mode and, eventually, lead to battery exhaustion. With the aim of promptly detecting firmware faults, there are diagnostic tools that help developers in either the development phase, or in the WSN deployment phase.

Tools that are used during the development phase usually require debugging interfaces, specific hardware implemented in the microcontrollers architecture, and Integrated Development Environments (IDEs) that allow for accessing the available functionality. Examples of these technologies are: the old JTAG interface used to program microcontrollers and to have access to special internal registers (e.g., hardware and software breakpoints); UART ports used for outputting log messages that are useful for debugging purposes; the EnergyTrace [66] technology, from Texas, that allows developers to measure the impact of firmware on nodes energy consumption; and the CoreSight [67] technology, implemented by ARM in their microcontrollers, which allows performance profiling, memory access, real-time tracing, and software debugging through a new type of interfaces, namely the Serial Wire Debug and the Serial Wire Output Pin (examples of variables that can be access using this type of technology are cycles per instructions, sleep cycles, and exceptions). On top of that, IDEs like Code Compose Studio (CCS) [65,68], among others, enable access to these tools and help developers to detect code faults.

None of the development phase diagnostic technologies are suitable for post-deployment because they require a wired connection between nodes and the tool’s hardware. Consequently, in the last decade, several deployment phase tools were proposed that are able to provide monitoring information, although with considerable limitations when compared to the functionality delivered by development phase tools. Some examples are [52], Nucleos [69], Enverilog [70], Marionete [60], Clairvoyant [71], NodeMD [72], L-SNMS [73], LIS [74], Memento [62], Dustminer [65], DT [75], Tracealyzer [76], and Dylog [51]. Despite their limitations, these tools allow WSN operators/managers to collect operating systems variables (e.g., task queue state, number of reboots), main application variables, and application events and states (like function calls). This information is usually stored in flash, RAM, or sent via the application main channel using logs. In order to deliver all of these without requiring an extra effort during the main application development, code instrumentation techniques are used, which make it possible to automate the process of adding firmware metrics collection and transmission functionality. Firmware data is usually sent using one of two paradigms: event-driven or query-driven. Additionally, some of the tools implement extra functionality, such as: source-level debugging, offering commands similar to hardware debugging (break, stop, watch, backtrace, etc.); remote call-specific functions; remote reprogramming; and specification of trace log events triggering conditions. Tools that usually read and write large amounts of data from/to flash memory (e.g., Tracealyzer) are not, in general, appropriated for industrial WSN technologies due to the energy wasted in the process.

### 3.3. Hardware Tools

Finally, a source of faults that diagnostic tools also commonly address is hardware faults. Hardware faults may occur before and/or after WSN deployment, and are due to one or more of several reasons, such as: bad hardware design, external environment phenomena, aging, and extreme temperatures. Hardware diagnostic tools can be divided in two groups: (1) external-physical-tools that are used by developers and operators; and (2) on-node-tools that infer hardware faults using available hardware resources.

The first group of tools consists of physical tools/equipment that operators and developers use to check hardware condition and the occurrence of faults. Tools like oscilloscopes, multimeters, and logic analysers are connected to the target system in order to check the condition of the electronics and the existence of faults. These tools are frequently used during the development phase, where developers search for electronics defects and for communication problems between hardware modules (e.g., using logic analysers [77]).

On-node-tools use a different approach. This type of tools is designed during the hardware and/or firmware development phase, with the specific purpose of collecting state information from the different modules using on-node components. In [78], the authors use the ARM CoreSight technology to create a WDP (Watch Dog Processor), by polling the main processor memory variables and setting conditions on these variables. Also using the same technology, in [79], the authors extend Hardware in Loop (HIL) tests, by incorporating some metrics provided by the CoreSight technology. Moreover, in [80], the authors propose a technique to read the energy wasted in boards that use switching regulators. By collecting these metrics, operators and developers are able to detect hardware faults that occur during the deployment phase without using external tools.

## 4. Proposed Architecture

Current WSN diagnostic tools have several drawbacks, as already pointed in our previous work [31]. In our opinion, some of these drawbacks are hampering the use of WSNs in industry because, nowadays, to the best of our knowledge, there aren’t multi-network, standard-compliant monitoring tools that support the IWSN technologies addressed in this proposal (ZigBee, ISA100.11a, WirelessHART, WIA-PA). In order to make this clear, in Section 2 and Section 3, a review of the state-of-the-art in what concerns the main Industrial IoT standards, network management, and diagnostic tools was made. As a result, some questions arose: how can multiple networks, possibly comprising equipment compliant with different standards, be monitored in an integrated way? How can management functionality be added at the gateway level? How can firmware and hardware be monitored without extra costs in hardware, firmware, and network?

In what concerns network monitoring, three important aspects were identified in Section 2 and Section 3: the set of techniques used by current diagnostic tools to monitor the network; the available metrics, provided by almost all industrial standards, that can be collected and shared at gateway level; and the current management standards. In what concerns hardware and firmware monitoring, several approaches and technologies were also presented, as well as log techniques that can be used to convey hardware and firmware metrics inside the network. Thus, the review made in these sections identified several crucial points and research directions that led us to the proposal of this architecture and related properties. Implementations of the proposed architecture will lead to diagnostic tools that are totally compatible with industrial standards. In this respect, a proof-of-concept implementation will be presented in Section 5, which also discusses evaluation results.

It should be highlighted that the current architectural proposal goes well beyond identifying components and respective abstract relations. More than defining the requirements, interactions, and roles to be performed by each architecture component, this section defines technologies, approaches, and protocols to be used in realistic, practical industrial scenarios. For instance, this section addresses the techniques and technologies for collecting hardware, firmware, and network metrics; defines the services used in each standard for conveying the monitoring information; and identifies the management protocols and technologies for dealing with the monitoring information.

The remainder of this section is organized as follows: initially, the proposed monitoring architecture is presented, providing the reader with a global view of its components and their main roles; subsequently, each component is described in detail, by specifying their role and requirements. Moreover, the technologies applicable to each component will be identified.

### 4.1. Architecture Overview

**Figure 1:**
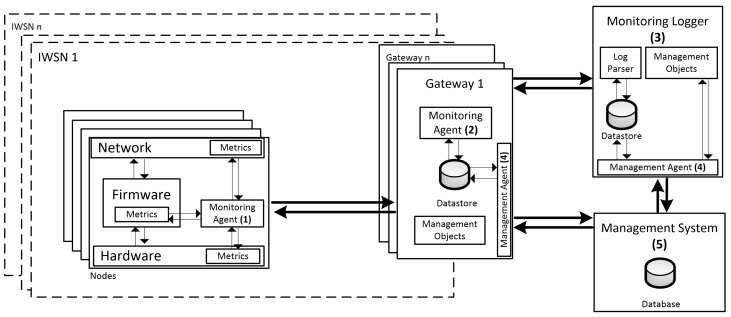
Proposed monitoring architecture.

With the emergence of several IWSN standards and the increase in the number of IWSN deployments, it is crucial to define an architecture able to monitor multiple-network, standard-compliant technologies. The proposed architecture, presented in Figure 1, represents a flexible, scalable, energy-efficient, low-cost, and multi-standard solution that covers hardware, firmware, and network monitoring. In order to ease the adoption by IWSN vendors, OEM producers, and developers, the architecture was designed according to six main guidelines: (i) it should support the monitoring of multiple IWSNs; (ii) it should support multiple IWSN standards; (iii) it should not lead to a significant increase in energy expenditure; (iv) the collection of hardware metrics should not increase the cost of manufacturing; (v) the acquisition of metrics should not have a significant impact on the main application size and delay, nor should it lead to a large traffic overhead; (vi) the network metrics defined by each IWSN standard should be used; and, lastly, (vii) the collection of sensor-node metrics (hardware/firmware) and network metrics should be independent. The proposed architecture has five base modules: (1) sensor node monitoring agent; (2) gateway monitoring agent; (3) monitoring logger; (4) management agents; and (5) management system.

The sensor node monitoring agent is responsible for the collection of node monitoring data (hardware and firmware metrics), and the sending of this metrics to the network gateway. The latter will forward the metrics to the monitoring logger, where they will be parsed and stored. The monitoring agent will use the most appropriate service in each industrial standard for forwarding the metrics. These metrics are encapsulated in a specific application format. During its operation (either when collecting information or when sending it), the monitoring agent should minimize the impact on the available resources.

Each industrial gateway has a pair of agents (one monitoring agent and one management agent). The gateway monitoring agent is the component that collects the network metrics and the gateway state (globally called management objects) and stores them in a local database (the datastore). These are then accessed in a standardized way by management systems, through the services delivered by the gateway management agent. In order to support the interoperability with management systems, the gateway also stores the representation of the management objects (i.e., IWSN standard metrics, and gateway state).

On the other hand, the handling of the collected sensor node monitoring data is carried out by a monitoring logger component, which parses the log messages and stores them in its datastore. The monitoring logger is a software component with two main sub-components: log parser and management agent. The log parser is the component that receives the log messages from the gateway, parses them, and stores them in the local datastore. Like the gateway, the monitoring logger locally stores a representation of the management objects. This representation enables the management agent to share the sensor node metrics with the management system in a standardized way (i.e., by using description languages such as SMIv2, XML e YANG). It should be noted that the monitoring logger is a logical component that can be deployed either on the gateway (if the manufacturer supports it) or on the management system.

The management system receives network monitoring data from the gateway management agent, and sensor node data (hardware and firmware metrics) from the monitoring logger management agent. Besides the traditional functions of configuring the monitoring capabilities of IWSN devices, the management system can include, for instance, a diagnostic tool that alerts operators or developers of critical events in the network, hardware, or firmware. Thus, the management system is capable of monitoring sensor nodes and network behaviour of multiple IWSNs using the standards addressed in this paper. This section outlined a proposal for a monitoring architecture for IWSNs that: does not require any modification to IWSN standardized technologies, benefits from the management information provided by each IWSN standard, and communicates with management systems in a standardized way. This addresses requirements (i), (ii), (vi) and (vii), identified in the beginning of this section. The next sub-sections detail each architecture component and show how the identified questions and requirements (especially, the ones more related with implementation strategies) are address by this proposal.

### 4.2. Sensor Node Monitoring Agent Overview

Sensor node metrics (hardware and firmware) are collected by the sensor node monitoring agent, as opposed to network metrics, which are collect by the gateway monitoring agent. Independence between the acquisition of network and sensor node metrics is one of the main features of the proposed architecture. This makes it possible to monitor the network independently from the acquisition of sensor node metrics. This separation also allows having closed modules in IWSN gateways (provided by IWSN vendors) and a more open monitoring logger module (that can be extended by developers if needed).

This section presents and discusses the main requirements for hardware and firmware metrics acquisition in the sensor node monitoring agent, and provides guidelines as to the technologies that can be used in this scope. Additionally, the approaches used in the transport of the logged data over the IWSN will be also presented.

#### 4.2.1. Hardware Metrics Collection

In order to persuade IWSM vendors and OEM producers to incorporate real-time monitoring in sensor nodes’ hardware, the monitoring techniques used by the sensor node monitoring agent should meet the following requirements: the added hardware components should not significantly increase the cost of sensor node manufacturing; the board space required by monitoring components should be minimal; the energy consumed by the hardware monitoring components should have a negligible impact on the battery life; processing and memory overhead should be minimum; and lastly, access to the hardware metrics should be seamless across all hardware platforms.

Regarding the latter requirement, considering the techniques presented in Section 3, those who enable direct access to microcontroller metrics (by using internal hardware registers and hardware counters) have a higher level of portability when compared to external physical tools. From the sensor node monitoring agent perspective, seamless access to hardware metrics, regardless the underlying technology, is extremely important. By using different Board Support Packages (BSPs) developed by hardware manufactures (e.g., Drivelib), or by using interfaces available in the sensor nodes operating systems (e.g., Contiki, RIOT), it is possible for sensor node monitoring agents to gather hardware metrics directly from each hardware platform. Hardware monitoring techniques that collect vast quantities of data to infer the hardware’s condition are not appropriate for industrial WSN application scenarios.

The work performed in [78,79,80,81] presents a set of metrics that can be collected with little cost and extra value to assess the health of hardware components. Specifically, in [80], the authors present a low-cost technique that, using an extra wire connected to an MCU hardware counter, enables the measurement of sensor nodes energy consumption. This technique was used in [81], in an anomaly detection system, with good results, and proved its low footprint in terms of memory, cost, and processing load. Using the same MCU technology (the MSP430 family), in [81], the authors present a technique that enables data acquisition on microcontroller cycles and execution time. In 16-bit architectures, the MSP430 family is one of the most used microcontrollers in WSN hardware due to its low power consumption. On the other hand, in 32-bit architectures, ARM microcontrollers are the most used ones. For these platforms, the work presented in [78,79] describes a set of metrics that can be collected from the CoreSight technology available in the ARM architecture. The Data Watch Point DWT block can provide several measurement statistics, such as interrupt overhead, and sleep cycles. Another block, the Embedded Trace Macrocell ETM, can provide statistics concerning the number of executed or skipped instructions.

Summing up, the monitoring of hardware condition can be performed at hardware level in compliance with the defined requirements. Furthermore, the proposed methods allow data acquisition for different types of microcontroller architectures and technologies. A set of techniques already addressed and proven by the scientific community were presented, and are recommended for use in the proposed architecture

#### 4.2.2. Firmware Metrics Collection

Development of firmware for sensor nodes can be made using one of two types of approaches: the “bare metal” approach or the OS-based approach. When the “bare metal” approach is used, the application is usually developed in C or C++, and access to the hardware is made by the BSP supplied by the manufacturer, resulting in a very hardware-dependent development. This type of development is less portable than OS-based development, in which hardware operations are managed by the OS. Developers only have to call OS generic functions that will, in turn, be translated into hardware-specific calls.

Firmware monitoring techniques used by the sensor node monitoring agent should support the monitoring of applications developed either using bare metal or OS-based approaches. Thus, as main requirements, firmware monitoring techniques should: be application independent; support, at least, C and C++ (according to [21] most OSs use these languages); be OS-independent; when enabled, have a negligible impact on the execution of the main application (i.e., a minimal increase in the microcontroller’s load); minimize the use of RAM, program memory, and external memory; be easy to integrate into the developers work flow; and not depend on physical access to the hardware. Similar to hardware monitoring techniques, firmware techniques that collect vast quantities of data and need physical access are not appropriate to this architecture.

From the set of techniques used in the tools presented in Section 3, instrumentation techniques are the only ones that fulfil the mentioned requirements. Tools that use instrumentation techniques with scripting languages allow developers to easily integrate them in their daily workflow and, at the same time, provide code tracing, debugging, profiling, and performance counters. Supporting instrumentation in C and C++ languages allows the use of instrumentation techniques in almost all OSs. In the proposed architecture, the sensor node monitoring tool collects firmware metrics by using instrumentation techniques.

In [51,52,53,74], the authors present some technologies, techniques, and metrics that use code instrumentation. For instance, in [74], the authors use the C Intermediate Language (CIL) to instrument the C source code. The instrumentation code is inserted by a parser before building the binary file. Using a similar approach, in [52], the authors use the PYCParser tool to instrument the code to support logging. On the other hand, in [50,51] the authors use binary instrumentation to inject the monitoring code using the trampoline technique. Differently from the other instrumentation techniques, in binary instrumentation, developers do not need to have access to the source code to change the program flow. Using trampoline techniques, the displaced instructions are executed and then another jump is made back to the actual code. This method allows the use of instrumentation in running code. These techniques allow the collection of several firmware metrics like function header, control flow (if, else, switch), function footer, function calls, variables values, and number of variable assignments.

In conclusion, from all the techniques presented in Section 3, solutions that need physical access to hardware interfaces (e.g., JTAG) to collect firmware monitoring metrics cannot be used in real industrial deployments. For this reason, the sensor node monitoring agent proposed in the current architecture collects firmware metrics using techniques deployed in the firmware by code instrumentation.

#### 4.2.3. Transport of Collected Data

IWSN standards were engineered to optimize four important sensor node resources: node battery, reliability, latency, and network bandwidth. In this type of networks, sensor nodes should operate during several years, and network resources should be optimized for conveying the collected data with a variety of QoS requirements. Typically, the transport of sensor node monitoring information has lower priority than the main application traffic because the collection of this information cannot compromise the sensor node main application (nor its operating lifetime). Thus, the transport of sensor node monitoring data made by the sensor node monitoring agent should fulfil the following requirements: the monitoring information should be sent using appropriate network services and without compromising the main sensor application; the monitoring information storage should be managed without compromising the main application (for instance using a FIFO buffer in RAM, and dropping data when the buffer is full); when available, data should have a network timestamp to make it possible to correlate several events in the network; when security is enabled for the main application, the monitoring data should also be secured; when log packets exceed the maximum payload size and no fragmentation and reassembly mechanisms are available in the underlying layers, the sensor node monitoring agent should support fragmentation and reassembly mechanisms; lastly, the impact of such operations on battery life should be minimal.

Regarding the timestamp requirement, from the set of the four analysed standards ZigBee is the only one that does not define a time standard in its specification. On the other hand, WirelessHART, ISA100.11a, and WIA-PA define their own time standard as Universal Time Coordinated (UTC), International Atomic Time (TAI), and UTC, respectively, and provide services to synchronize sensor nodes [9]. Consequently, in ZigBee, sensor node monitoring data cannot be correlated without implementing additional mechanisms in the sensor nodes that enable for synchronizing the network time between nodes.

As already mentioned, the Maximum Transmission Unit (MTU) in IEEE802.15.4 is 127 bytes. Thus, all of the four standards use fragmentation and reassembly mechanisms to overcome this limitation. WIA-PA and ISA100.11a implement this mechanism at the network layer, unlike ZigBee that implements it at the application support sub-layer. WirelessHART also supports the transport of large packets, but, in this case, sensor nodes need to allocate a special type of transport service—the block data transfer. This service only allows the existence of a unique block transfer service for the whole network [9,12]. Thus, in WirelessHART, the transport of blocks of data that exceed the maximum MTU size requires fragmentation and reassembly to be implemented at application level.

Another important aspect is the security of sensor node monitoring data. All of the industrial standards within the scope of this architecture offer hop-by-hop and end-to-end encryption. In the cases of ZigBee, WirelessHART, and ISA100.11a, security is managed using a centralized approach. On the other hand, WIA-PA uses a distributed approach, where the security manager together with the routing devices configure the security measures and forward the keys to field devices. All the standards under consideration provide encryption using symmetric keys, although ISA100.11a can also use asymmetric encryption in device joining processes. Last but not least, the highest level of IEEE 802.15.4 security policy is AES-128-CCM. Consequently, most IEEE802.15.4 radio chips support AES-128-CCM operations at the hardware level [9].

In order to minimize the impact on the main application traffic, the approach to transporting sensor nodes monitoring data should be carefully selected for each standard, as presented below.

In WirelessHART, data transport can be done using one out of four types of services: block transfer service, maintenance service, periodic service, and event service. The block transfer service can only be used by one sensor node at a time. Thus, this service is not appropriate to transport sensor node monitoring data. The maintenance service provides the network with a minimal bandwidth for basic sensor nodes control and management operations. Because this is a low bandwidth service, it cannot handle the transport of sensor node monitoring data. The event service is used by applications to send data packets during unexpected events, such as alarms and warnings (when this service is requested, the radio needs to define the latency associated to the service) [35]. Finally, the publish service is used to periodically send data, like sensor readings (when this service is requested, the radio needs to define the interval for sending the data) [82]. Taking into consideration the characteristics of each service, we conclude that the most appropriate service to transport the data is the publish service, since the event service is a service used by applications with latency constraints, and the monitoring data does not have such requirement.

ZigBee does not specify services for data transport, leaving this to the application layer. According to the ZigBee specification [12], in order to guarantee compatibility among different manufactures, ZigBee devices need to implement application profiles (also called endpoints). An endpoint is a ZigBee application for a specific domain with a set of clusters (or application messages) that can be mandatory or not. In turn, clusters are composed by attributes that represent the exchanged data. ZigBee sensor nodes that implement the same ZigBee profile (endpoint) are able to communicate with each other. In the context of this architecture, the sensor node monitoring agent is an endpoint and a set of cluster messages that can be sent to the ZigBee coordinator (that must also support the same endpoint).

Compared to the other standards, ISA100.11a is by far the most complex and customizable standard (see Table 4). Instead of using the term services, used in WirelessHART, in ISA100.11a, contracts establish the resources allocated by the system manager to devices. Before a device can send data to another device, a contract needs to be created. Contracts are identified by an ID, unique within the scope of the device (but not necessarily so in the scope of the network), and are unidirectional. The system manager is the device that has the authority to assign, modify, and revoke contracts. Like WirelessHART services, there are several types of contracts and attributes that can be used for establishing service levels. Firstly, contracts may be of two types: periodic, that schedule network resources for the periodic sending of data; or, otherwise, non-periodic. Secondly, contracts can also be negotiable. The system manager may change or revoke the contract to make resources available to other high priority contracts. Lastly, contracts can have several levels of priorities: best effort queued, used in client-server communications; real time sequential, used in voice and video applications; real time buffer, used for periodic communications; and network control, used for managing network devices by the system manager. Message retransmission can, additionally, be enabled or disabled inside the contract. Thus, sensor node monitoring agents that run inside an ISA100.11a network should request a non-periodic contract type, which can be negotiated and revoked if needed. In this way, the system manager can revoke the contract of the sensor node monitoring agent, thus guaranteeing that the main sensor application can deliver sensor data without disruption. Additionally, the contract priority used by the sensor node monitoring agent should have the best effort queued type.

In the case of WIA-PA, networks may operate in two distinct modes: a hierarchical network topology that combines star and mesh, or a star-only topology. The star-only topology is a special case of the hierarchical network. For this reason, this topology uses the same services available in the hierarchical topology for data transport. In WIA-PA, the Virtual Communication Relationship (VCR) is the main standard block to access the objects specified in the User Application Objects (UAOs). VCRs distinguish the routing and communication resources allocated to each UAO. Each VCR has a VCR identifier, a source UAO ID, a destination UAO ID, address of source device/destination device and the VCR type. Similar to WirelessHART services and ISA100.11a contracts, in WIA-PA, VCRs can be classified according to the application scope: publish/subscriber (P/S) VCRs, used for publishing periodic data; report source/sink (R/S) VCRs, used for transferring aperiodic events and trend reports (alarms); and client/server (C/S) VCRs, used for transferring aperiodic and dynamic paired unicast messages (for getting and setting operations in UAO). Additionally, VCRs also provide aggregation methods. In this architecture, the sensor node monitoring agent data are UAOs capable of representing log messages. Thus, sensor node monitoring agents, available in each field device, as well as routing devices, need to select the appropriate VCR. P/S VCRs are appropriate for real-time operations, using exclusive timeslots in intra and inter-cluster communication. For this reason, this type of VCR should not be used for sending the monitoring data. On the other hand, R/S and C/S VCRs use the CAP period of the IEEE802.15.4 slot inside clusters, and use shared timeslots in inter-cluster operations. Thus, C/S and R/S VCRs are the most appropriate for transporting sensor node monitoring data in WIA-PA networks.

### 4.3. Gateway Monitoring Agent

The gateway monitoring agent is the component in charge of representing management objects data in a standardised way, and of sending network monitoring data to the management systems. This agent also deals with data from multiple standards. The gateway monitoring agent is independent from the sensor node monitoring agent, thus allowing the separation of sensor node data collection from network monitoring data coleection. In this context, gateway monitoring agents must meet the follow requirements: network monitoring should not significantly increase the cost of equipment nor the cost of installation; collecting network metrics should add low overhead to the gateway; monitoring should be energy-efficient; the monitoring solution should be easy to install and should be extensible; and, lastly, gateway monitoring agents should support widely used industrial standards, namely the ones addressed in this paper.

Considering the three types of techniques identified in Section 3.1 (active, passive, and hybrid), active type techniques are the only ones that are easy to install and do not rely on extra hardware to perform monitoring tasks. However, these types of techniques usually consume sensor node resources, as pointed in Section 3. On the other hand, in Section 2, the revision made to the metrics shared between nodes showed us that the standards under consideration can support the sharing of critical information related to network and sensor node state, which can be used for resource allocation and routing tasks. Thus, by using these metrics, active tools will be capable of fulfilling the identified requirements without spending significant sensor node resources. Furthermore, some current industrial solutions available on the market already provide these metrics at the gateway, using proprietary libraries. The only disadvantage of active type techniques is the partial coverage in ZigBee networks because some metrics cannot be gathered by the coordinator. This arises from the fact that, in ZigBee, route computation is done in a distributed fashion for some topologies.

### 4.4. Monitoring Logger

As shown in Table 1, the standards under consideration use different application approaches. Specifically, ZigBee, ISA100.11a, and WIA-PA use object-oriented representation, whereas WirelessHART uses a command-oriented representation. Sensor node monitoring data needs to comply with the representation defined in each standard, which leads to different representations for the same sensor node data. To solve this issue, the monitoring logger is the component that parses distinct standard representations into the same representation.

The monitoring logger should fulfil the following requirements: support the connection to the different standards “gateways”; support the parsing of the distinct application protocols using a sub-component (the log parser); support the representation of sensor node monitoring data in the languages supported by the management protocols; allow access to the monitoring data by management systems, using the management agent; and, lastly, store the sensor node monitoring data in a datastore. As a software component, the monitoring logger can be installed as an extra software module in industrial gateways or in another compatible equipment.

### 4.5. Management Agents and Management System

Management systems are one of the building blocks of current IP-based networks. By monitoring the networks and their equipment, operators and manufactures can maximize the network uptime, improving the delivered quality of service. When applied to IWSNs, network and sensor node monitoring can also provide similar benefits. It is unthinkable to have a large IWSN that uses one or more standards without a central management system to perform predictive maintenance of sensor nodes. As presented in the previous sections, hardware, firmware and network monitoring data can be delivered by the sensor node monitoring agent and by the gateway monitoring agent, respectively. However, a key to the puzzle of this architecture is missing. To improve interoperability between these agents, different manufactures, and diagnostic tools, appropriate management protocols and syntax languages are needed. In this context, management agents should fulfill the following requirements: agents should allow the representation of sensor node and network data as managed objects; when needed, managed objects should be able to be extended, supporting additional monitoring metrics; management agents should be able to share management object information with management systems, updating the model used in management systems when needed; the management protocol should be able to support security mechanisms; management agents should run in IWSN gateways, for which the minimum hardware requirements should be set to IETF Class 2 [20] (e.g., gateways that can support, for instance, a simplified version of Linux-based operating system); management protocols should support notification messages (query-based architectures can be heavy for IWSN gateways and are not scalable); lastly, the management agent must operate in IP networks (IWSN gateways must be able to be connected to an IP network).

When network management technologies were previously analyzed in Section 2, some protocols, syntaxes, and key features were identified. Current network management protocols can be divided into protocols designed for resource-constrained devices, and protocols for traditional networks. While traditional network management protocols are more mature and widespread, resource-constrained management protocols use more advanced, simpler, and modern technologies for representing management objects (e.g., YANG), and for data transport (e.g., CoAP, HTTP). Here, we highlight that protocols designed for resource-constrained devices can also be used in more powerful devices, like IWSN gateways. However, there are other aspects that need to be addressed regarding the requirements defined in this section. From the set of syntaxes for representing management models, XML and YANG are the most flexible and most simple languages. The learning curve to describe management objects using SMIv2 is longer than using languages like YANG and XML. Thus, SMIv2 is not recommended in the scope of this architecture. Consequently, SNMP is also not recommended for this architecture. Moreover, when working with data models, one important requirement is the sharing of the model with management systems. From the remaining protocols proposed in the scope of this architecture, NETCONF [41], COMI [47], RESTCONF [46], and LwM2M [39] allow the discovery of the models and of the resources available in each management node, by using discovery commands or specific URIs that enable to retrieve the used models. Additionally, other important requirement is the use of notification messages. By creating subscriptions to different management topics, diagnostic tools will be able to have the monitoring information in real time and without extra effort, contrary to what happens in query-based protocols that need to actively look for the monitoring information. Considering the set of analysed management protocols, the only one that does not support notifications is the NETCONF protocol. Before a client and a server can exchange management messages, NETCONF has to establish a permanent connection using SSH and TCP. Thus, it is impossible to notify management clients in the case of a connection loss. RESTCONF, LWM2M, and COMI have support for notification messages.

Last but not least, one of the main characteristics of protocols for resource-constrained devices is the capability to use the protocols in the node itself (something they were developed for). However, in IWSNs, for which application protocols are statically specified by the standard, it is not possible to use other protocols in sensor nodes, such as the management protocols we’ve been addressing. The only option is to represent managed objects in the gateway of each standard. The authors of [83] present a solution to monitor and manage legacy systems with LWM2M protocol, by using several LWM2M clients to represent each legacy device behind the gateway. Additionally, as presented in [84], using the YANG language it is possible to represent the network metrics shared with a gateway in a WirelessHART network. In this way, COMI and RESTCONF can also be used in the gateway to represent legacy devices.

## 5. Proof-of-Concept

In the previous section, the monitoring architecture was presented as well as its components, requirements, and available solutions for each component in light of the existing standards and protocols. Being a general monitoring architecture that integrates several network standards, hardware technologies, and firmware architectures, a proof-of-concept implementation of such architecture showing each supporting technology and monitoring technique is unfeasible. Thus, in this section, a proof-of-concept scenario is presented using a small testbed with a WirelessHART network. Using some of the technologies and solutions already presented in the Section 3 and Section 4, we set out to prove that the proposed architecture is able to monitor the network and the sensor nodes (hardware and firmware) operations, with low impact on IWSN resources.

This section starts by presenting the test scenario, comprising the industrial application, and the deployed hardware and firmware components. Secondly, the collected metrics and associated mechanisms are presented. Thirdly, data acquisition and processing of sensor node metrics are explained. Lastly, the impact on the network and sensor node resources is analysed and discussed.

### 5.1. Test Scenario

**Figure 2:**
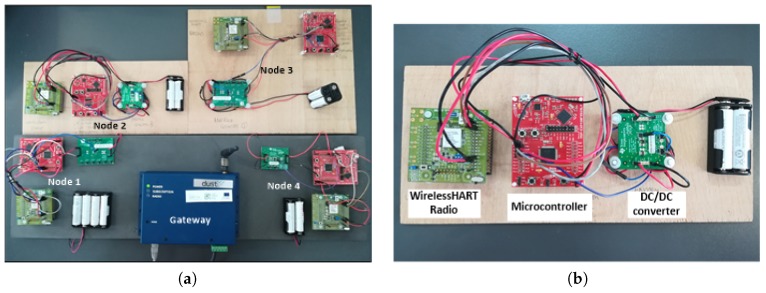
Validation of the monitoring architecture using a WirelessHART testbed. On the left (**a**) the Gateway and four sensor nodes, on the right (**b**) the sensor node components.

The proposed monitoring architecture was tested using a typical industrial application. The proof-of-concept scenario consisted of four sensor nodes and a WirelessHART gateway. On one hand, sensor nodes monitor the temperature of the industrial assets and send their readings to the gateway. On the other hand, the gateway controls all the traffic in the network, performing the role of a network and security manager, Figure 2a. According to the proposed monitoring architecture, WirelessHART sensor nodes and the gateway are capable of sharing sensor node monitoring data and network metrics.

The firmware deployed in the sensor nodes can be divided into two distinct modules: the industrial application, and the sensor node monitoring agent. The industrial application is responsible for temperature sampling and for controlling the network operations (by sending control messages to the radio, such as network joining, network notifications, and service requests). On the other hand, the sensor node monitoring agent collects hardware and firmware metrics and sends them by using a specific network service.

As presented in the sensor node monitoring agent description, Section 4.1, WirelessHART defines four types of service level agreements: block transfer service, maintenance service, periodic service, and event service. The proof-of-concept application running at the microcontroller requests the following services to the gateway: a publish service, for sending temperature data every minute; an event service, for sending alarms if the temperature rises above a certain threshold (the verification is made every 30 s); an additional publish service that is requested and deleted each time the sensor node needs to send monitoring data (every 15 min); and, lastly, a maintenance service, directly allocated by the radio that handles all the control messages between the radio and the network manager.

In terms of hardware, the sensor nodes are formed by three components, Figure 2b: radio, microcontroller, and power supply. The radio handles the communication tasks with other network nodes and with the microcontroller (Linear DC9003A-C). The microcontroller runs the industrial application and the sensor node monitoring agent (Texas MSP430F5 launchpad, Texas Instruments, Dallas, TX, USA). Lastly, the power supply is formed by a pack of batteries and a DC/DC converter used to supply energy to the radio and the microcontroller. Two of the nodes use TPS62740EVM-186 buck converters (Texas Instruments), and the other two use TPS61291EVM-569 boost converters (Texas Instruments). By using these DC/DC converters, we can measure the energy expenditure of each sensor node almost for free and in real time.

The gateway (LTP5903CEN-WHR, Analog Devices, Norwood, MA, USA) controls all network operations, manages the network, performs security operations and, at the same time, connects the IWSN with the industrial network through an IP network. Additionally, an application running at the gateway allows the subscription of specific types of messages (e.g., application messages, sensor node monitoring data messages, and network report messages). This application performs the role of the gateway monitoring agent, collecting the reports identified in Table 2.

Finally, the monitoring logger was developed as a python application that implements the log parser sub-component. When the monitoring logger application starts, a specific subscription is made to the network manager in order for it to receive the sensor node monitoring messages. After that, when the data arrives at the sub-component, the log parser converts the packets to JSON objects and stores them in a datastore.

In this proof-of-concept scenario, we only intend to measure the impact of the architecture on sensor node resources (memory, processing, energy) and on the network operation (overhead in relation to the sensor node traffic). Thus, we did not implement the management agents presented in the architecture (at the gateway and at the monitoring logger). Such agents were already presented and are freely available, as described in [85,86], and, thus, their implementation in this proof-of-concept scenario would not provide relevant added-value. However, we should emphasize that the WirelessHART network metrics management models that would be required for such implementation were already created by us and are available for download in [84].

### 5.2. Collected Metrics

The architecture proposed in this paper allows the collection of hardware, firmware, and network metrics using agents deployed in sensor nodes and network gateways. In Section 4, some of the requirements were presented, as well as some monitoring techniques already explored by other researchers. Thus, to prove that this architecture has low impact on sensor nodes resources, our test scenario implements some of these techniques, enabling the collection of metrics from the hardware of the Texas MSP430F5 launchpad, from the industrial application, and from the WirelessHART network.

Collecting hardware metrics was done using techniques that directly access the registers and counters of the Texas MSP430F5 launchpad. As this is a 16-bit microcontroller, the implemented techniques were the ones presented in [80,81], comprising a processing metric, an energy metric, and a time metric. The processing metric gives the number of instructions executed by the microcontroller. Using switching regulators in combination with the technique presented in [80], it was possible to have an energy metric that provides the energy spent by sensor nodes. Lastly, the time metric gives a time measurement in milliseconds.

Specifically, the metrics were implemented in the following way. Firstly, there are two possible approaches to implementing the processing metric in the Texas MSP430F5 launchpad: (1) by configuring pin P7.7 to output the MCLK clock and connecting it to a hardware counter; or (2) by using the same clock source and same frequency of the MCLK clock in SMCLK, and configuring a counter to count it. As the Texas MSP430F5 launchpad does not give us access to pin P7.7, the second approach was used, and TimerA was configured to be sourced by the SMCLK. Secondly, the energy metric was obtained by connecting the output of the inductor used in the switching regulator (used as a DC/DC converter, either the TPS62740EVM-186 or the TPS61291EVM-569) to the TimerA clock signal input (P1.6) of the microcontroller. Lastly, the time metric was obtained using the TimerB, configured to be sourced by the ACLK (being sourced by ACLK allows to count time even during sleep periods such as Low Power Mode 3 (LPM-3)).

Apart from hardware metrics, the architecture also enables firmware monitoring by using instrumentation techniques applied to the main application code. In order to prove that it is possible to collect some metrics with low impact on the sensor node resources, manual instrumentation of the code was performed, with the objective of obtaining a trace of function calls. Specifically, the function calls trace was obtained by assigning a specific ID to each function in the code. Additionally, the instrumentation code enabled each executed function to collect the function identifier, the function duration, the spent energy, and the processing load (by using the metrics collected from the hardware). The details of the data processing and transport over the network will be presented in the following sub-section. Lastly, after collecting the metrics concerning the sensor node state (hardware and firmware), the gateway monitoring agent collects the reports identified in Table 2, namely device health report, neighbour health list report, neighbour signal levels, and network alarms.

### 5.3. Sensor Node Instrumentation and Monitoring Information Processing

The proof-of-concept implementation presented here aims at demonstrating the applicability of the proposed architecture to several software development scenarios. Specifically, the architecture should support OS-based applications as well as “bare metal” applications, as in the current case. With this in mind, the application was developed based on a function-queue-scheduling software architecture. Similar to what happens in OS-based approaches, the functions are added to a queue of function pointers, and called when appropriate. The scheduler is responsible for getting the next function in the queue, which will then be executed. On completion, the microcontroller is put in low power mode (LPM) to save energy.

**Figure 3:**
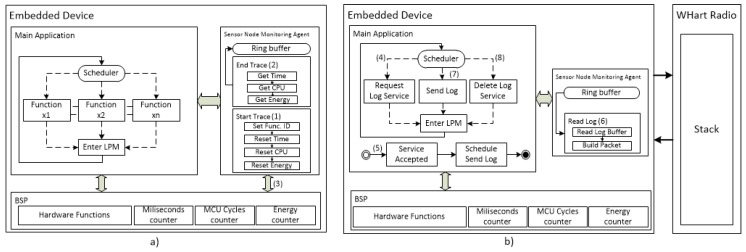
On the left, (**a**) the application architecture and the sensor node monitoring agent acquiring the state information. On the right, (**b**) the request of the WirelessHART publish service.

The main topic of this section is, nevertheless, the sensor node monitoring agent and its interactions with the BSP, the main application, and the network (Figure 3). The sensor node monitoring agent is a library developed in C/C++ that collects the sensor node state data, stores it, and triggers a periodic event to send the monitoring data over the network. By manually inserting a simple call to the sensor node monitoring agent at the beginning of each function to be monitored (see Figure 3a (1)), it is possible to record the state of the sensor node’s firmware and hardware. The monitoring information is then stored in a ring buffer (Figure 3a (2)), until it is sent over the network. In addition, the hardware state data is collected directly by the sensor node monitoring agent library from the BSP (Figure 3a (3)).

Last but not least, as illustrated in the Figure 3b, when there is sufficient data to fill an IEEE 802.15.4 data packet payload, the sensor node monitoring agent requests a publish service to send the data available in the ring buffer (4). If the network has enough resources to send the data at the requested rate, an authorization is received (5), and the sending process starts (6–7). In case the network does not have enough resources, the microcontroller will receive a service denial, and the sending of the log will be postponed. When no more data is available in the ring buffer, the sensor node monitoring agent requests the deletion of the service in order to free the network resources (8).

### 5.4. Results

In order to show that the proposed architecture can be implemented in industrial scenarios with efficiency and low impact on resource consumption, some experiments were conducted, whose results are described in this sub-section.

The performed tests can be divided in two groups: tests that measure the impact on the sensor node resources, and tests that measure the network operation overhead introduced by the sensor node monitoring agent. It should be noted that, as presented in Section 2, the industrial standards addressed in this paper already share network metrics to perform routing and other network related operations and, thus, these metrics do not add extra overhead to the network operation. Hence, the only network overhead is the one caused by sensor node monitoring agents.

**Figure 4:**
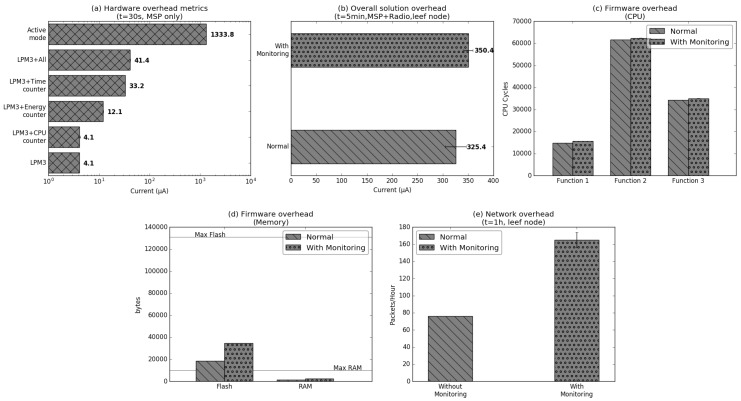
Obtained results.

For the first group of tests, the used hardware and software were, respectively, the Texas Instrument MSP-FET (with EnergyTrace technology support) and the Code Composer Studio (CCS, version 7, Texas Instruments) connected directly to the launchpad debug pins and bypassing the board debugger. Each test was performed during 30 s and repeated 10 times. In the second group of tests, the gateway monitoring agent was used to collect the impact of the traffic generated by the sensor node monitoring agent on the network, by collecting device health reports. In these tests, we collected health reports over a period of 10 h. The results presented in Figure 4 show (a) the impact of the hardware techniques on the sensor node battery lifetime; (b) the overall impact of the sensor node monitoring agent on the sensor node lifetime; (c,d) the impact of the sensor node monitoring agent on the microcontroller processing and memory, respectively; and (e) the overhead caused by the transport of the sensor node monitoring data over the network.

In order to measure the impact of the hardware monitoring metrics on sensor node resources, a simplified version of the industrial application was developed with the objective of guaranteeing the independence of each added metric. In this test, the energy expenditure was measured by the MSP-FET. Additionally, the radio was disconnected in order to allow the easy detection of the comparatively small increase of energy expenditure caused by each hardware metric.

As shown in Figure 4a, the energy spent by the MSP430 in LPM3 without any hardware metric enabled is around 4.1 μA. Enabling the processing metric does not increase the energy spent by the microcontroller. In this LPM, the MCLK and the SMCLK are turned off and, consequently, the processing metric interrupt will not occur when the microcontroller is in LPM. On the other hand, the energy counter increases the microcontroller energy expenditure in LPM because, even in this LPM mode, the system consumes energy and, as a result, the interrupt associated with the energy counter mechanism will be executed (this interrupt is tied with an hardware counter that is sourced by an external clock). In this mode, when enabled, the microcontroller counter consumes 12.1 μA (σ = 0.00 μA). Lastly, the time counter, sourced by the ACLK consumes around 33.2 μA (σ = 0.05 μA).

Contrary to the significant increase in energy consumption in LPM associated with each metric, the energy spent by the complete solution in active mode with the radio on (acquisition plus transport of the monitoring data) only corresponds to a 3.5% increase (Figure 4b) in relation to a non-monitoring situation. Using two AA NiMh batteries (1.2 v, 1900 mA), the lifetime of the sensor node is 658 days (σ = 6 days) without monitoring and 635 days (σ = 12 days) with the sensor node monitoring agent enabled.

The latency generated by adding the monitoring mechanisms to the sensor nodes firmware, as well as the microcontroller’s memory taken by the monitoring mechanisms are also two relevant aspects in assessing a monitoring solution. The firmware graphs, in Figure 4c,d, show the number of cycles executed by the microcontroller in three firmware functions, and the RAM and ROM requirements, respectively, with and without monitoring. In terms of processing overhead, the manual instrumentation performed in the code of each function inserts an average of 758 cycles (σ = 3 cycles), adding 93 μs of latency at 8 Mhz clock. As can be seen in (c), the overhead is quite small, when compared to the cycles spent to execute the functions without monitoring, representing 5.1%, 1.2%, and 2.2%, for functions 1, 2, and 3, respectively. This test was performed manually, by measuring the difference of cycles at the beginning and at the end of the instrumentation functions, using the Code Composer and the MSP-FET. An analysis in terms of memory was also made, measuring the increase of flash memory and RAM. In what concerns flash memory, the main application without monitoring occupies 18.590 KB, as represented in Figure 4d. Adding the sensor node monitoring agent and the instrumentation code, the application size increases to 34.659 KB. This represents an increase of 12.26% of the total capacity of the flash memory in this microcontroller version (131.072 KB). Lastly, as also presented in Figure 4d, the main application without monitoring takes 1.631 KB of RAM. Adding the sensor node monitoring agent to the application increased RAM usage to 2.506 KB. When compared to the initial utilization, the sensor node monitoring agent only consumes an additional 8.54% of all the RAM available in the microcontroller (10.240 KB).

The network overhead caused by the sensor node monitoring agent was also measured. Using health reports collected every fifty minutes by the gateway monitoring agent, we were capable of measuring the traffic generated by a leaf node in normal operation and with the monitoring capabilities enabled. As can be seen in Figure 4e, the main application sends 76 packets/hour (σ = 0 packets) on average. Most of this traffic is generated by the publish service that sends the temperate values every minute. Enabling the sensor node monitoring agent causes a traffic increase of 46%, sending an average of 165 packets per hour (σ = 9 packets), of which 89 packets are generated by the sensor node monitoring agent. In WirelessHART, the IEEE802.15.4 payload is limited to 94 bytes per packet. Thus, each packet sent by the sensor node monitoring agent is capable of carrying six log entries, each with log id, function id, function duration, processing metric, and energy metric.

Summing up, the assessment of the proposed monitoring architecture, carried out through this proof-of-concept implementation, shows its low impact and high efficiency in what concerns sensor node resources and network. Table 5 summarises the obtained results. By causing a mere 3.5% reduction in sensor nodes’ battery lifetime, introducing a 93 μs latency overhead in each function, occupying 12.26% of flash memory, and using 8.54% of RAM, the implementation enabled the monitoring of hardware and firmware in sensor nodes. Despite the increase of 46% in network traffic generated by sensor nodes, the proposed solution, here implemented for WirelessHART, used network resources in a smart way. The monitoring data was only sent when the network had resources to send it, requesting the service and deleting it each time the sensor node monitoring agent needed to send monitoring data. Using this approach, the sensor node monitoring agent only requested free network resources, not used by the main industrial application.

## 6. Related Work

The solution presented in this paper proposes a multi-domain architecture that is able to monitor the condition of IWSNs from different perspectives. To the best of our knowledge, the proposed architecture is the first of its kind. The related work reviewed in this section also addresses the multi-domain nature of the architecture, by analysing the contributions in several domains. In Section 3, part of this multi-domain analysis was already made, in a detailed way, when different techniques to monitor the network, firmware and hardware were presented. On the other hand, this related work section will provide a broader overview, by analysing solutions from the Industry 4.0 perspective.

Current related work in the Industry 4.0 topic is extensive. In general, most of the existing monitoring architectures target the prevention of security attacks in ICS systems, although without taking into consideration the IWSN technologies analysed in this paper [87]. Some of these solutions have been proposed because of the paradigm shift that occurs nowadays in the ICS core, with the introduction of IP networks. Additionally, more recent technologies such as Software Define Networks (SDN) [88] and Cloud-based platforms [89] are being considered in such architectures, due to the rich network metrics and flow control mechanisms that these technologies make available. However, this type of control and monitoring is specific for wired IP networks, that can work in a decentralised manner, and with different application requirements in mind (e.g., troughput). However, as presented before, applications in industrial proccess automation need deterministic latency, instead of pure throughput. This is, by way of example, the kind of requirement being considered by the Time-Sensitive Task Group (TSN). The group analyses the possibility of deterministic services in IEEE 802 based networks. As consequence, technologies like SDN cannot be applied to IWSN standards like WirelessHART, ISA100.11a, WIA-PA and ZigBee, at least inside the wireless network domain, at this stage, due to their centralized and deterministic nature. Solutions like [88] can only be used at gateway level, by virtualizing some of the non-deterministic features and roles in another device (e.g., the security manager in WirelessHART). Thus, in this section, only solutions proposed for the industrial standards addressed will be considered.

From the first perspective, the authors of [90,91] propose techniques that improve the reliability of the network at routing schedule-level: by using a finite-state Markov model [90], and by specifying scheduling emergency and recovery mechanisms when a path-down alarm occurs [91]. On the other hand, the work presented in [92,93,94] proposes several techniques and platforms that can be used to monitor some IWSN. In [92], the authors develop a passive monitoring tool for evaluation of the deployed WirelessHART networks using passive sniffers deployed in the network area. Additionally, in [93], the authors propose an hybrid monitoring technique to monitor wireless and wired industrial technologies in the scope of the project HiFlecs. Lastly, in [94], the authors present a debugging tool that is able to collect in the SensorLab2 environment routing topology information and end-to-end performance, by debugging the information using UART ports and then converting it to TCP/IP messages. Finally, some authors also show some concerns in the lack of integration solutions between the industrial standards [95,96,97]. In [95] the authors presented how the MQTT protocol can be used together with ISA S5.1 and ISA95/88 standards to represent the monitoring and control in topics using the URI scheme. Lastly, in [96] the authors presents how the WirelessHART standard and the ISA100.11a standard can be integrated using the Modbus protocol and a proprietary software called Wonderware InTouch.

When comparing the reliability solutions already proposed by other researchers with the architecture proposed in this paper, some differences appear. Most of the work performed in this field is not taking into consideration the main components that may produce faults in an IWSN (firmware, hardware and network). Additionally, most part of the work focus in a specific problem like [90,91], and do not address the needs of monitoring several process automation networks at the same point. Additionally, some of the works that present solutions to monitor these standards [92,93,94] keep proposing passive monitoring tools that need extra networks to be installed, adding extra costs to a technology designed to be low-cost. Lastly, most of the work [95,96,97] in the field of integration focus their effort in the representation of the sensor data, and any of them try to explore the use of management protocols like LWM2M and COMI (protocols specific for constrained devices) to represent and make available the monitoring data.

## 7. Conclusions

The need for more dynamic and capable solutions in the Industry 4.0 field has contributed to an increase in the use of IWSN technologies in industrial applications over the last few years. As is widely recognised, WSN technologies have a considerable number of advantages over traditional solutions, although they are not free from drawbacks, namely in what concerns reliability, due to their resource-constrained nature. Nevertheless, hardware and firmware of sensor nodes are becoming more mature and capable. On one hand, the hardware is becoming more efficient and, at the same time, hardware manufactures are introducing new features in their architectures that allow the collection of state metrics. On the other hand, the adoption of operating systems in WSNs is also growing, enabling the development of firmware architectures and tools capable of collecting firmware state metrics. In a broader context, at the same time, ICS networks are shifting from networks disconnected from public infrastructures to systems connected to the Internet. This transformation is also exposing these systems to a new set of security threats, and new types of IDS systems are needed. To keep up with all of these changes and, at the same time, improve IWSN reliability, new network monitoring architectures are needed, more specifically, architectures that are not only capable of monitoring IWSNs from a network perspective, but also from the hardware and firmware perspective.

Such a multi-domain IWSN monitoring architecture was proposed in this paper, considering the main process automation IWSN standards. In order to back up the proposal, a comprehensive revision of the state of the art was needed, in order to introduce the different concepts in each domain. This was done in Section 2 and Section 3, by surveying WSN diagnostic tools and the relevant industrial technologies, and by presenting several network, hardware and firmware metrics as well as the techniques used to collect and transport them over wireless networks. Additionally, being a multi-network monitoring architecture, several management models were also presented. Lastly, the architecture was presented in Section 4, and validated in a real testbed scenario, in Section 5. In Section 4, when the architectural components were described, some possible solutions were presented in order to provide some guidelines for implementation. Nevertheless, as shown by our proof-of-concept implementation, the architecture can be used to perform the monitoring of a variety of sensor and network-wide parameters, in a way that does not increase the cost of node manufacturing, does not have a significant impact on sensor node resources, and does not steal network bandwidth from the main application.

By proposing such architecture and by analysing relevant techniques and technologies, more questions and future research directions arrose. Thus, as future work, we plan to further explore the proposed monitoring architecture by extending and refining the existing implementation. In the firmware domain, we aim at extending the architecture in order to provide support to more advanced instrumentation techniques, such as those proposed in [52,53,74]. On the other hand, in what concerns hardware monitoring, we would like to extend the compatibility to the ARM architecture, by supporting some of the metrics available in the CoreSight technology. From the network perspective, we would like explore new ways of reducing the overhead created over the network. Lastly, we plan to extend the implementation to other technologies (e.g., ZigBee, WIA-PA and ISA100.11a). Finally, by using the proposed architecture, we also plan to explore the architecture’s data collection potential for building a diagnostic tool, and to share the acquired datasets with the community.

References

## Figures and Tables

**Table 1:** Summary of main features, adapted from [9] and [18].

Layer	ZigBee	WirelessHART	ISA100.11a	WIA-PA
APP	Object-oriented; Profile Defined Protocol	Command-oriented; HART protocol	Object-oriented; Native Protocol; Multi-wired field bus protocols	Object-oriented; Profibus/FF/HART Protocol; Virtual Device
APS	Discovery of New Device; Binding; Fragmentation/Reassembly	-	Basic Tunnelling; Smart Tunnelling	Data Communication and Management Services
TP	-	Block Data Transfer; Reliable Stream Transport; Convergence	Optional Security Features; Connectionless Services	-
NWK	Tree Routing/AODV Routing; Address Assignment; Network Joining/Disjoining	Graph/Source/Superframe Routing	Addressing (6LowPAN); Routing Address Translation; Fragmentation/Reassembly	Addressing; Static Routing; Fragmentation/Reassembly
DL	-	Slot Timing Communication; Time Synched TDMA/CSMA; Channel Hopping	Grapgh/Source/Superframe Routing; Slot Timing Communication; Duocast Transaction; Time Synched TDMA/CSMA; Channel Hopping	Frequency Hopping; Aggregation and Disaggregation; Time Synchronization
MAC	IEEE 802.15.4 Mac Layer	IEEE 802.15.4 Mac Layer (partially implemented)	IEEE 802.15.4 Mac Layer (partially implemented)	IEEE 802.15.4 Mac Layer
PHY	IEEE 802.15.4 PHY 868 M/915 M/2.4 GHz Radio Data rate: 20 Kb/; 40 Kb/s; 250 Kb/s	IEEE 802.15.4 PHY 2.4 GHz Radio Data rate 250 Kb/s	IEEE 802.15.4 PHY 2.4 GHz Radio Data rate 250 Kb/s	IEEE 802.15.4 PHY 2.4 GHz Radio Data rate 250 Kb/s

**Table 2:** IWSN Network metrics.

Standard	Network Reports	Metrics	Report Type	Visibility
ZigBeePRO	Network Status	No route available; Tree link failure; Non-tree link failure; Low battery level; No routing capacity; No indirect capacity; Indirect transaction expiry; Target device unavailable; Target address unallocated; Parent link failure; Validate route; Source route failure; Many-to-one route failure; Address conflict; PAN identifier failure; Network address update; Bad frame counter; Bad key sequence number	Event	Coordinator or Routers
	Link Status	Neighbour network address; Incoming cost; Outgoing cost	Periodic		
	Network Report	Radio channel condition; PAN ID conflict	Event /Periodic		
WirelessHART	Device Health	Packets generated by device; Packets terminated by device; DL mic failures; NWK mic failures; Power status; CRC errors	Periodic	Network Manager
	Neighbour Health List	Total number of neighbours; Nickname of Neighbour; Mean RSL; Packets transmitted to the neighbour; Packets received from the neighbour; Failed transmissions	Periodic		
	Neighbour Signal Levels	Total number of neighbours; Nickname of neighbour; RSL of neighbour in DB	Periodic		
	Alarms	Path Down (Nickname of the neighbour); Source Route Failed (Nickname of the neighbour and NWK MIC from the NPDU failed routing); Graph Route Failed (Graph Id of the failed route)	Event		
ISA100.11a	Connectivity Alert per Channel	Attempted unicast transitions for all channels; Percentage of time transmissions on channel x did not receive an ACK); Percentage of time transmissions on channel x aborted due to CCA	Periodic	System Manager
	Connectivity Alert per Neighbour	RSSI level; RSQI level; Valid packets received by the neighbour; Successful unicast transmissions to the neighbour; Unsuccessful unicast transmission; Number of unicast transmissions aborted (by CCA); Number of NACKS received; Standard deviation clock correction	Periodic		
	Neighbour Discovery Alert	Total number of neighbours; Neighbour address; Neighbour RSSI; Neighbour RSQI	Periodic		
WIA-PA	Path Failure report	Route ID	Periodic	Network Gateway
	Device Status report	Number of sent packets; Number of received packets; Number of MAC layer mic failures detected; Battery level; Number of restarts; Uptime since last restart	Periodic		
	Channel Status report	Channel Id; Neighbour device address; LQI; Channel packet loss rate; Channel retransmission count	Periodic		
	Neighbour Reports	Neighbour address; Backoff counter; BackoffExponent; Last time communicated; Average RSL; Packets transmitted; Ack packets; Packets received; Broadcast packets	Periodic	

**Table 3:** Network management protocols revision.

Standard	Modeling Language	Supported Operations	Resource Constrained Devices Support	Notifications Support	Used Protocols
SNMP	SMIv2	Monitoring/Configuration (not used to configure devices)	No	Yes	UDP
NETCONF	YANG	Monitoring/Configuration	No	No	SSH/SSL/HTTP
RESTCONF	YANG	Monitoring/Configuration	Yes	Yes	HTTP/TLS/TCP
COMI	YANG/SMIv2	Monitoring/Configuration	Yes	Yes	CoAP/DTLS/UDP
LWM2M	XML/YANG	Monitoring/Configuration/ Application Management	Yes	Yes	CoAP/DTLS/UDP and SMS

**Table 4:** Network transport revision.

Standard	Time Standard	Fragmentation and Reassembly	Security	Service Level Agreement (Available Options) Support	Service Level Agreement (Recommended) Protocols
ZigBee	-	Application Sub-Layer	Symmetric encryption available in all standards and supported by IEEE802.15.4 hardware	-	-
WirelessHART	UTC	Block transfer service		Publish, Event, Maintenance, and Block Transfer service	Publish service
ISA100.11a	TAI	Network Layer		Contract type: periodic and non-periodic Contract priority: best-effort queued, real-time sequential, real-time buffer and network control	Contract type: Non-periodic Contract priority: Best-effort
WIA-PA	UTC	Network Layer		Publish/subscribe VCRs, source/sink VCRs, and client/server VCRs	Source/sink VCR or Client/server VCR

**Table 5:** Evaluation results overview.

Metric	Value
Node lifetime	−3.5%
Overhead latency	93 μs
Flash overhead (each instrumented function)	+13 bytes
RAM overhead (each instrumented function)	+14 bytes
Flash usage	* 12.26%
RAM usage	* 8.54%
Network traffic	+46%

* when compared with the max capacity available.
